# Study of Aramid Yarns Sizing

**DOI:** 10.3390/polym14040761

**Published:** 2022-02-15

**Authors:** Katarina Krstović, Stana Kovačević, Ivana Schwarz, Snježana Brnada

**Affiliations:** Department of Textile Design and Management, Faculty of Textile Technology, University of Zagreb, Prilaz baruna Filipovića 28a, 10 000 Zagreb, Croatia; stana.kovacevic@ttf.unizg.hr (S.K.); ivana.schwarz@ttf.unizg.hr (I.S.); snjezana.brnada@ttf.unizg.hr (S.B.)

**Keywords:** sizing with pre-wetting, sizing without pre-wetting, sizing of aramid yarns, mechanical yarn properties, laboratory-sizing machine

## Abstract

The process and efficiency of sizing aramid yarns before the weaving process was studied. The sizing was carried out under different conditions, with and without the pre-wetting of the threads before the actual sizing process. Two groups of yarns were tested. The first group consisted of five yarn samples that were blended with 95% meta-aramid and 5% para-aramid in counts of 20 × 2, 17 × 2, 14 × 2 and 12.5 × 2 tex. The second group of yarns consisted of three yarn samples that were blended with 93% meta-aramid, 5% para-aramid and 2% carbon in counts of 20, 20 × 2 and 17 × 2 tex. The inlet moisture of the yarn before sizing was 40% (with pre-wetting) and 4% (without pre-wetting), and the outlet moisture after drying was 4%. In order to carry out such tests to reproduce them, the sizing was carried out on a laboratory-sizing machine with the possibility of adapting to industrial conditions. According to the obtained results related to the properties of yarn before and after sizing, it can be concluded that sizing of aramid yarns is justified. When sizing the yarn without pre-wetting, the mechanical properties improved, especially breaking force, strength and abrasion resistance. Irregularity and hairiness were also reduced, especially when sizing with pre-wetting. Yarn hairiness or the frequency of protruding fibres also decreased with sizing in almost all samples and sizing conditions. The second group of yarns with a carbon fibre content mostly showed better mechanical properties before sizing, which continued after sizing. In general, the aramid yarn sized with pre-wetting showed certain deformations caused by stretching in the wet state and thus reduced the size pick-up, which caused less breaking forces and strength. Sizing with pre-wetting resulted in a slightly better smoothness of the thread and its higher evenness. It can be concluded that the aramid yarn should be sized with a lower size percentage (up to 4.5%), i.e., without pre-wetting in order to minimise the deformation of the yarn during sizing and thus improve the mechanical properties in the weaving process.

## 1. Introduction

New textile materials made of new raw material compositions of fibres and yarns, whose properties can be significantly changed depending on the intended use and conditions, require an increasingly complex approach to processing up to the production of the end product. Each new fibre represents a significant unknown in further processing into the final product, and blending with other fibres becomes an even bigger and more comprehensive problem in carrying out the processing operations correctly [1,2,3,4].

The fibres for protective fabrics production are selected primarily according to the properties necessary to meet certain aspects of protection. Woven fabrics for protective clothing must meet, in addition to the primary, some other criteria: abrasion resistance, resistance cracking when bending, resistance to extreme temperatures, resistance to tearing, puncture, ignition and flame resistance and guaranteed minimum tensile strength, etc., which depends on the intended purpose of the garment [5].

During the technological process of making fibres, yarns and then woven fabrics, they come into contact with different chemical agents and mechanical stresses in different conditions, which affects the final quality of the finished fabric. Their largest deformations occur in the weaving process, primarily as a result of high dynamic forces in extremely short cyclic pulses, and extremely aggressive friction of threads on the elements of the loom. In order to reduce the inevitable consequences that occur in the weaving process, warp threads usually need to be sized beforehand. Despite the fact that some threads meet the strength and resistance to abrasion, they often need to be sized to smooth and protect the surface structure of yarn and fibres, reduce the occurrence of permanent deformation and reduce the occurrence of static electricity. This paper will investigate the justification of sizing aramid and carbon yarn in the woven fabric preparation processes [6,7,8].

The objective is to damage the fibres, the yarn and later the fabric as little as possible through the processing procedures and to make the end product as cost-effective and environmentally friendly as possible. Unfortunately, it is not always possible to retain or improve the properties of fibres and yarns and go through the processing procedure to the fabric production. Mechanical and wet processes for fibres, yarns and fabrics cause frequently irreparable damages, which are unfortunately expected and planned. The objective is to reduce the damage caused in most cases by exceptional cyclical stress and abrasion (rewinding of the yarn, warping and weaving) and chemical processes (sizing, dyeing, bleaching and mercerising, etc.) [9,10,11,12].

Sizing in warp preparation for weaving is an extremely complex process that is constantly being developed in terms of plant automation, sizing conditions and the use of sizing agents. Due to their complexity in simultaneously sizing thousands of threads, unwinding them perfectly, passing them through the line in a dry-wet-dry condition and winding them in an extremely tensioned condition, deformation of the threads occurs and is alleviated by reducing the thread tension in the segments where the threads are wet [13,14,15,16,17,18].

To study the effectiveness of the sizing of newer raw materials and materials, it is necessary to monitor important parameters before and after sizing under different sizing conditions and to qualitatively find the best and most economical method. In order to achieve repeatability of the test and the most favourable sizing procedure for the corresponding yarn types in this work, a laboratory-sizing machine was used, on which the sizing conditions can be adapted to industrial ones. It is well known that the objective of sizing is to reduce the number of yarn breaks and deformations in the weaving process, thus increasing fabric quality and machine efficiency. The rheological properties of the sizing, the physical-chemical properties of the yarn as well as the technological parameters of the machine depend significantly on sizing quality. Sizing quality depends primarily on the intensity of adhesiveness, i.e., the adhesion between the fibres in the yarn and the sizing agent, so that this sizing agent can reinforce the fibres and take on the cyclic stresses that occur during the weaving process. Sizing quality depends not only on the intensity of the adhesion between the sizing agent and the fibres, but also on the ability to form a surface film on the yarn. The surface film protects the fibres primarily from abrasion and cyclic stress [19,20,21,22].

Sizing without pre-wetting is a common process that allows a good surface size pick-up with full or partial penetration of the size between the fibres. The threads enter the size box without pre-wetting with low moisture content but with air saturation in the fibre interstices. By pressing the air out of the yarn shortly before the yarn is immersed in the size, the space between the fibres remains partially filled with air, which offers a certain resistance to the penetration of the size into the yarn interior and comes to a standstill on the yarn surface. This dry sizing procedure is favourable for the yarn, which is sensitive to stretching in the wet state. In sizing without pre-wetting, the size penetrates more slowly into the fibre interstices in the yarn; the viscosity and concentration increase so that the size is retained more strongly in the first yarn layers and on the yarn surface, creating a surface film. The size is easier to remove after drying in the weaving process because it is not sufficiently bonded to the yarn on the surface and its efficiency is lower compared to wet sizing [23,24,25,26,27].

Wet yarn sizing, i.e., sizing with pre-wetting, is still new and rare in the sizing process. This sizing process is an insufficiently researched area, especially when using new raw materials. With this type of sizing, the yarn enters the sizing box with higher moisture content than in the standard sizing. The contact of the retained water in the yarn results in a bond with the size, which allows faster and easier penetration into the yarn, i.e., diffusion of the size into the interior of the yarn. This method achieves a stronger bond between the size and the yarn, less removal during weaving, higher elasticity and greater economy, which in some cases gives preference to this sizing method [28,29,30,31,32].

The objective of this research was primarily to investigate the efficiency of sizing aramid yarns, and the difference between sizing with and without pre-wetting. Due to the relatively low moisture regain of aramid and carbon fibres and their synthetic origin with a closed and smooth surface obtained by chemical spinning, the adhesion with sizing agents is not as intense as with natural fibres, but sizing in this case flattens fibres and glues them together.

Due to the relatively low moisture absorption of aramid and carbon fibres and their synthetic origin with a closed and smooth surface obtained by chemical spinning, the adhesion with sizing agents is not as intense as with natural fibres, but the sizing in this case smooths the fibres and glues them together. Due to the sensitivity of these fibres in the wet and tensioned state, the sizing results nevertheless support sizing without pre-wetting.

According to the previous findings, the sizing of aramid yarn has not been investigated. The justification for the sizing of aramid yarn in a mixture with carbon fibres is not known, although their use is increasing, especially for high-performance protective clothing. This research will conduct yarn sizing in the standard way without pre-wetting and with a new pre-wetting process with yarn, which provides broader and more detailed information in the segment of weaving warp preparation for the latest fibre types, as well as new yarn and woven fabric structures.

## 2. Materials and Methods

### 2.1. Yarn

The investigation was carried out on 7 different types of yarn, the basic properties of which are listed in Table 1. The first group of samples (1–4) has the same raw material composition and differs only in fineness and number of twists. Samples 5 to 7 represent the second group of samples with a different raw material composition, differing only in fineness and number of twists.

### 2.2. Sizing

#### 2.2.1. Sizing Agents

For the investigation carried out, sizing agents and their percentage in the size were selected which are used in the industry for the above-mentioned yarn counts, i.e., which best suit the materials investigated (yarn type, i.e., fibres) and the needs and requirements of the size and the subsequent weaving process.

The following sizing agents were used: Fibrosint C75 (Pulcra Chemicals GmbH; synthetic polymer) and Inex 773C (Pulcra Chemicals GmbH; chemical composition: polyvinyl alcohol) in the percentages listed in Table 2.

#### 2.2.2. Sizing Equipment

**Figure 1:**
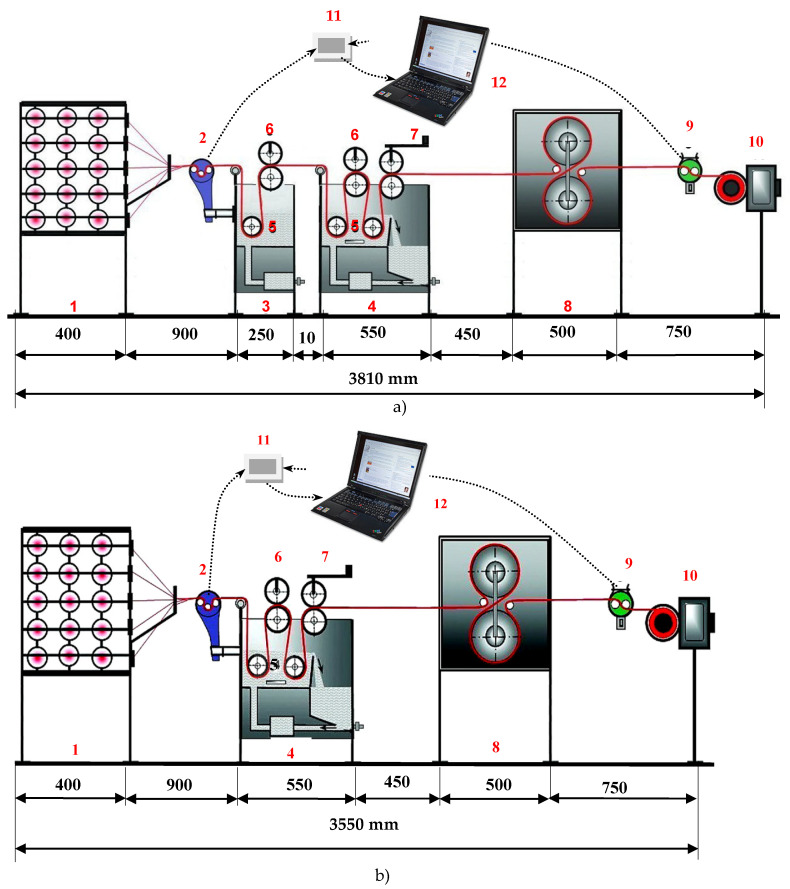
Laboratory-sizing machine (constructed in the Faculty of Textile Technology, University of Zagreb, Croatia); (**a**) sizing with pre-wetting and (**b**) sizing without pre-wetting; 1—creel for cross wound bobbins, 2—thread tension measuring device, 3—pre-wetting box, 4—size box, 5—rollers for immersing yarn into water and size, 6—rollers for water and size squeezing, 7—regulation of the pressure of the last squeezing roller, 8—contact dryer, 9—moisture contact measuring device, 10—winder of the sized yarn, 11—converter and 2, 9, 11, 12—measuring equipment.

Sizing conditions were adapted to industrial conditions, and sizing was performed on the sizing machine shown in Figure 1 [33,34]. In order to be able to adjust the sizing conditions more easily and cost-effectively, a laboratory-sizing machine was used, on which the samples for these investigations were made. Two sizing processes were used: the standard sizing process without pre-wetting with an inlet moisture of 4% and a newer sizing process with pre-wetting with an inlet moisture of 40%. It is possible to carry out both processes on the laboratory-sizing machine constructed at the University of Zagreb Faculty of Textile Technology (acknowledged as consensual patents under the numbers PK20070247 and PK20070248 at the State Intellectual Property Office of the Republic of Croatia).

During the sizing process, the sizing unit consists of two boxes, a pre-wetting box for dipping the yarn in warm water (3) and a size box (4). The unit is 3810 mm long. When sizing without pre-wetting, the pre-wetting box is removed and the unit is much shorter, i.e., 3550 mm. Sizing conditions are the same for both sizing processes (with pre-wetting and without pre-wetting). The thread tension is individually adjusted via the built-in brakes on the creel (1) and equalised before sizing. The temperature of the water in the pre-wetting box (60 °C) and the size solution in the box (80–85 °C) is kept constant by the built-in thermostats and heaters, which indirectly heat the water and size solution through the walls of the box. The circulation of the size solution in the size box (4) is made possible by the built-in pump. The force of squeezing the size solution at the exit from the size box is made possible using the lever mechanism (7), with which the size pick-up is directly adjusted. The sized yarn is dried in contact with two heated rollers (8). The sizing machine has the possibility to adjust the sizing speed (10). By installing measuring probes (2 and 9), converters (11) and computers (12), the machine enables continuous measurement of thread tension (55 cN) before dipping into the box (2) and contact measurement of the thread moisture (4%) at the exit of the dryer (9). The sizing speed of the thread is 3 m/min, the pressure of the last pair of rollers for squeezing out the excess size solution is 8.5 N/cm^2^ and the temperature of the cylinders in the dryer for contact drying of the thread is kept constant at 110 °C. After drying, the yarn is wound on a winch. The first and last 10 m of the sized yarn were not taken for further testing to ensure steady conditions (size solution and water temperature, drying temperature, speed, tension and outlet moisture).

#### 2.2.3. Sizing Conditions

In the sizing process, all conditions affecting the size pick-up were kept constant (Table 3), so that equal conditions were achieved and maintained throughout the thread-sizing process. The yarn samples were sized simultaneously with 5 threads from each sample at a length of 220 m. During sizing, the yarn tension at the entry of the box and the moisture of the yarn at the exit of the dryer were continuously monitored.

### 2.3. Mechanical Properties of Yarn

The mechanical properties of the yarn represent the basic parameters required for further analysis of the properties relevant to the weaving process.

**Figure 2:**
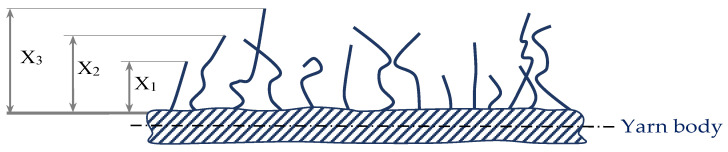
Measurement of yarn hairiness using USTER^®^ TESTER, principle of optical hairiness testing; X_1_, X_2_, X_3_, …, X_n_—distances measured.

- The breaking properties of the yarn were tested according to ISO 2062, on the Statimat M Textechno tensile tester, which includes the following properties: breaking force F (cN), elongation at break ε (%) and breaking strength (cN/tex) and work to rupture (cN × cm).
- Uster hairiness index (H)—total length of protruding fibre/cm length of yarn. It is also termed as the H-value and was tested on Uster Tester 4, Zellweger Uster, Switzerland, ISO 2649. USTER^®^ TESTER with the hairiness metre attachment measures the hairiness index [30]. A parallel beam of infrared light is illuminated on the yarn as it runs past the measuring device, as depicted in Figure 2. Only the light that is scattered by the protruding fibres from the main body of the yarn reaches the detector. The hairiness measurement is done by the amount of scattered light that is converted to an electric signal by the apparatus. Therefore, this instrument monitors only the total hairiness. The testing speed of this instrument is 800 m/min. The measuring principle is based on evaluation with diffracted light. The hairiness value H is defined as the total length of all fibre ends sticking out in cm, related to a yarn section of 1 cm in length. The device gives information on the standard deviation of the hairiness value H and provides a hairiness diagram and spectrogram.

**Figure 3:**
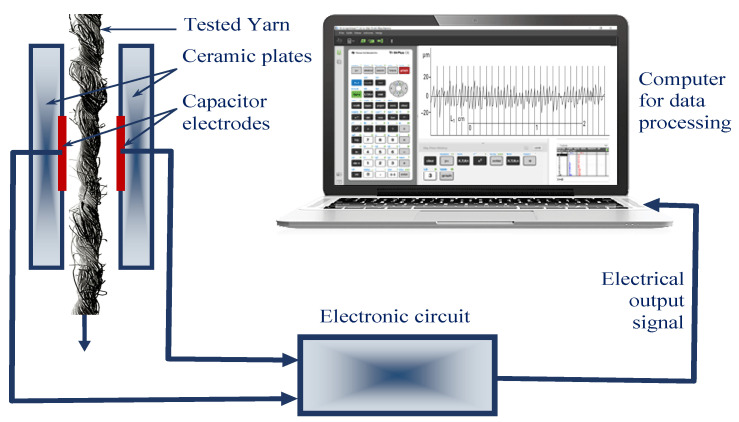
Capacitance principle of Uster Tester.

Yarn irregularity or coefficient of variation of mass (U/%) was tested (with capacitive method) on Uster Tester 4, Zellweger Uster, Switzerland, ISO 2649. The Uster yarn evenness tester is the instrument developed by the Switzerland Uster Company, and it is used to test the evenness of yarns. This evenness tester uses the capacitance conversion principle 3 to translate the non-electric yarn section change into the electric singles representing the change of section [35,36]. The testing part is composed by the capacitors with two parallel metal plates. Because the dielectric coefficient of the fibre materials exceeds the dielectric coefficient of air, when the sample of yarn enters into the capacitor with certain speed, the capacitance of the polar plate will increase and the change of the capacitance is related with the actual volume of the yarns in the polar plates (Figure 3).

- Yarn twist was determined using a MesdanLab Twist Tester according standard ISO 17202.
- Abrasion resistance (A) (number of cycles)—the abrasion resistance test was performed on the Zweigle G551 tester, where each of the 20 threads was simultaneously subjected to the abrasion process under a load of 20 g until the thread breaks. By moving the roller coated with 600 grit emery paper along the length of the thread and rotating it around its axis, a certain intensity of abrasion of thread and emery paper is achieved. During abrasion, the thread weakens and at the moment when the weight mass hung on the thread overcomes the strength of the thread, a break occurs and the number of movements of the roller (n) is registered until the break of each thread.
- The moisture content of the yarn after drying was measured with a contact moisture meter (K.P. Mundinger GmbH, type Aqua-boy), which was redesigned for continuous measurement and storage of the data in the computer.
- The yarn tension was measured with a tension meter (Schmidt, model ETM), which was redesigned for continuous measurement and storage of the data in the computer.
- The gravimetric method was used to determine the quantity of the size. Prior to the sizing process, the samples are dried to absolutely dry, after which the samples are weighed and then returned to climatic conditions and sized; after sizing the samples are dried again to absolutely dry and weighed. Size pick-up (*Sp*) on the yarn was determined using the mass technique according to the Equation (1):

(1) $$Sp=\frac{G_{S}\;\left(g\right)-G_{U}\;\left(g\right)}{G_{U}\;\left(g\right)}\;\;\left(\%\right)$$

*Sp* (%)—amount of the size pick-up,
*G_S_ (g)*—mass of the absolutely dry sized yarn,
*G_U_ (g)*—mass of the absolutely dry unsized yarn.

## 3. Results and Discussion

The tests carried out revealed very interesting results, which were confirmed and supported by the analyses, comparisons, indicators of variability, degrees of error, final evaluations and statistical data processing carried out.

**Figure 4:**
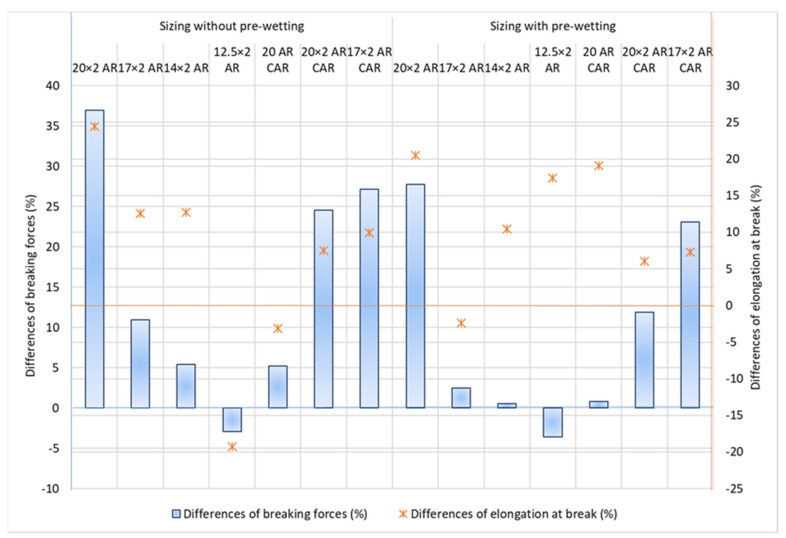
Differences in breaking forces (F; cN) and elongation at break (ε; %) after sizing without pre-wetting and after sizing with pre-wetting.

**Figure 5:**
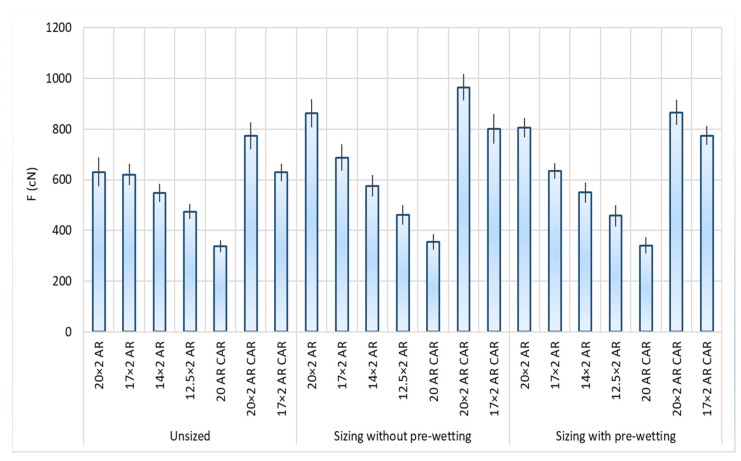
Breaking force and error size depending on yarn samples and sizing conditions.

**Figure 6:**
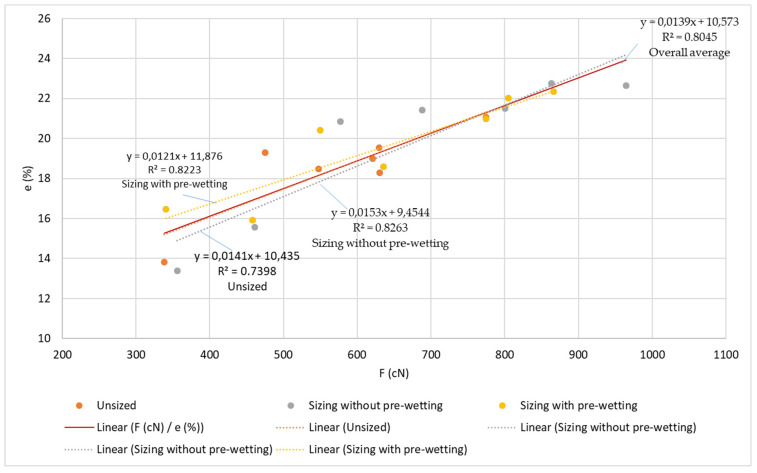
Regression lines, regression equations and correlation coefficients between breaking forces and elongation at break for the unsized yarn, sized yarn and the overall average.

**Figure 7:**
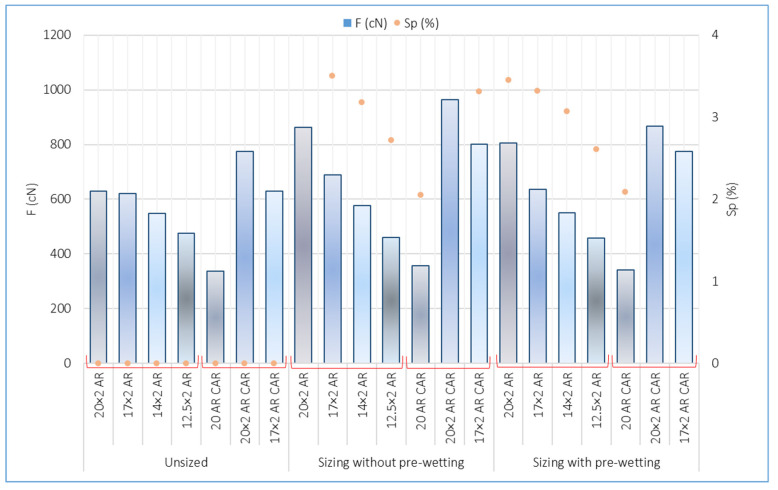
Beacking force (F (cN)) snd size pick-up depending on sizing conditions and samples.

**Figure 8:**
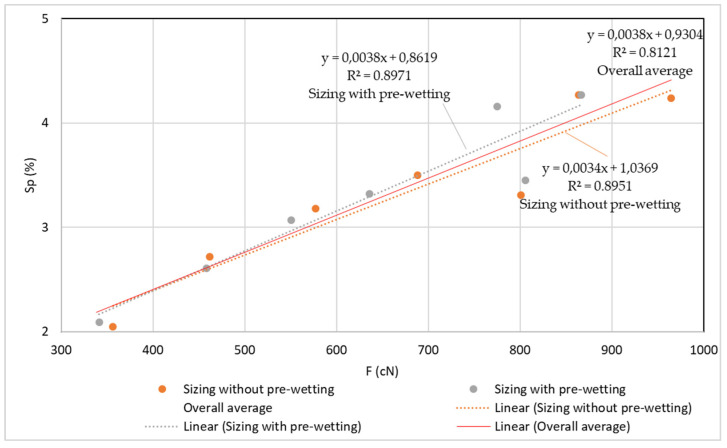
Regression lines, regression equations and correlation coefficients between breaking forces (F; cN) and size pick-up (Sp; %) for the unsized yarn, sized yarn and the total average.

**Figure 9:**
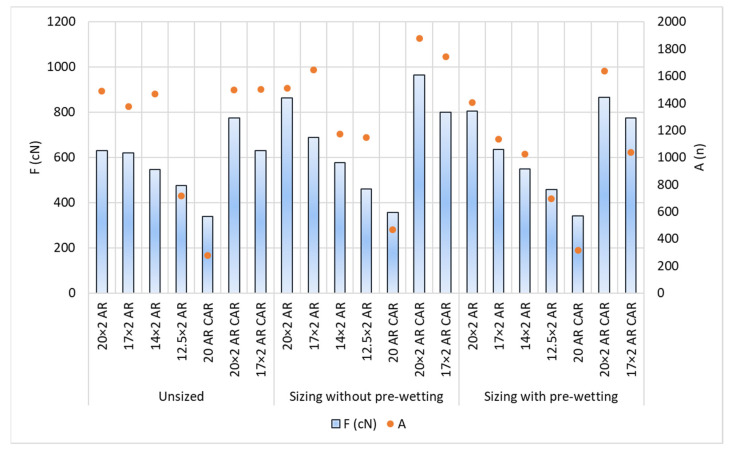
Breaking force (F; %) and abrasion (A; n) depending on sizing conditions and samples.

**Figure 10:**
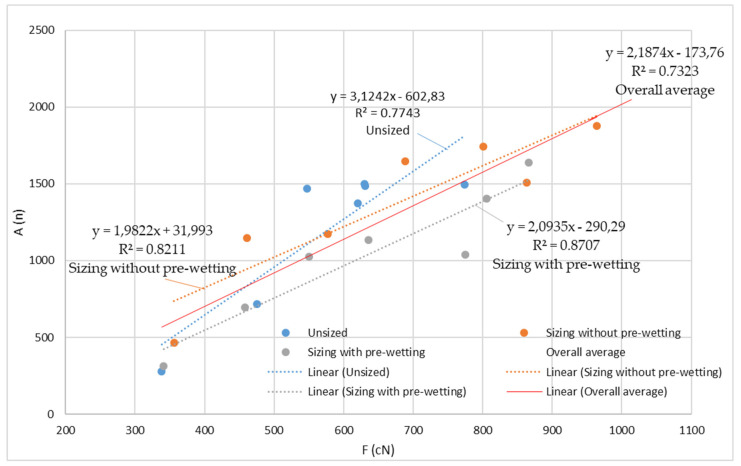
Regression lines, regression equations and correlation coefficients between the breaking forces (F; cN) and abrasion resistance (A; n) of the unsized yarn, sized yarn and the overall average; n—number of cycles of movement of the roller along the length of the yarn until breaking.

**Figure 11:**
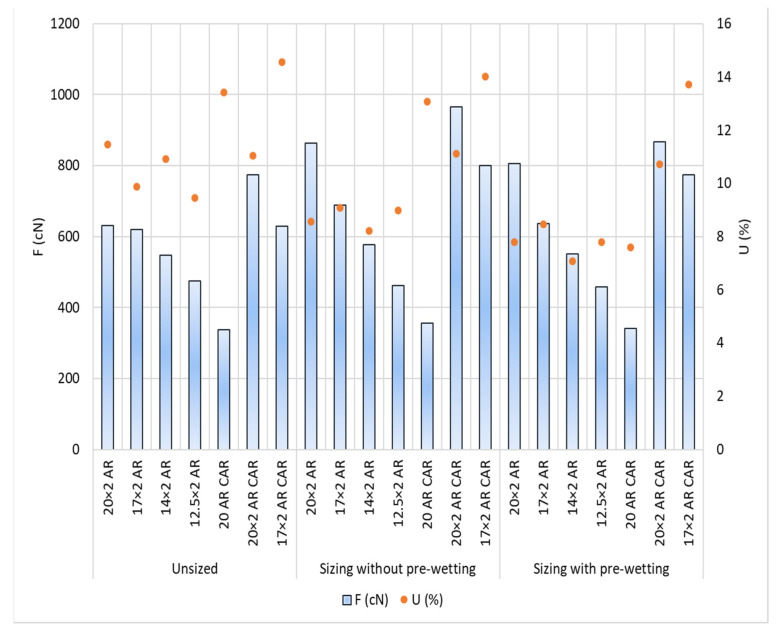
Breaking force (F; cN) and yarn irregularity (U; %) depending on sizing conditions and samples.

**Figure 12:**
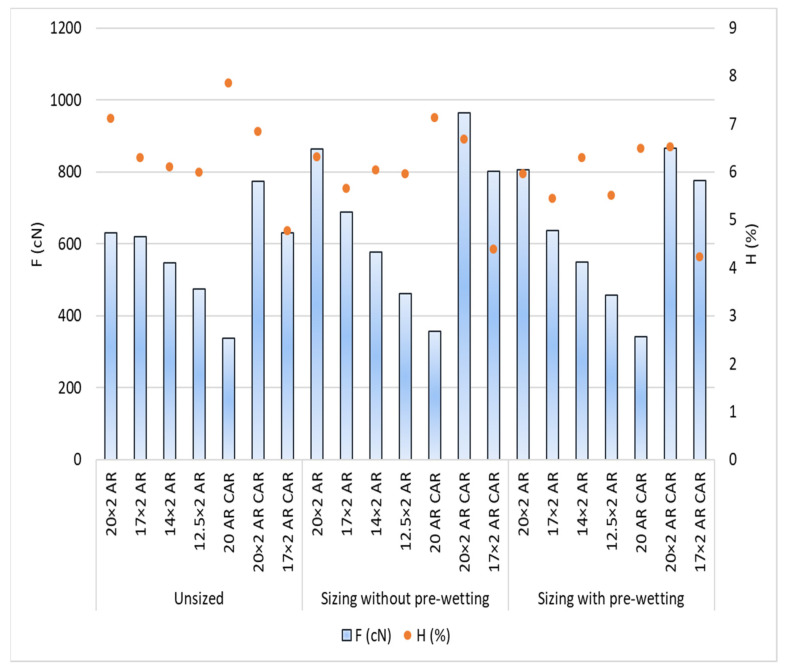
Breaking force (F; cN) and yarn hairiness (H; %) depending on sizing conditions and samples.

**Figure 13:**
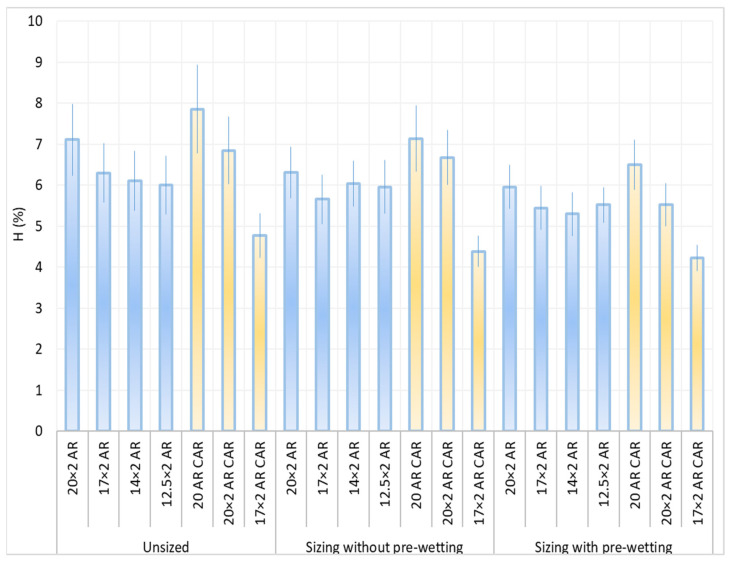
Yarn hairiness (H; %) depending on the sizing conditions and samples with a coefficient of error.

**Figure 14:**
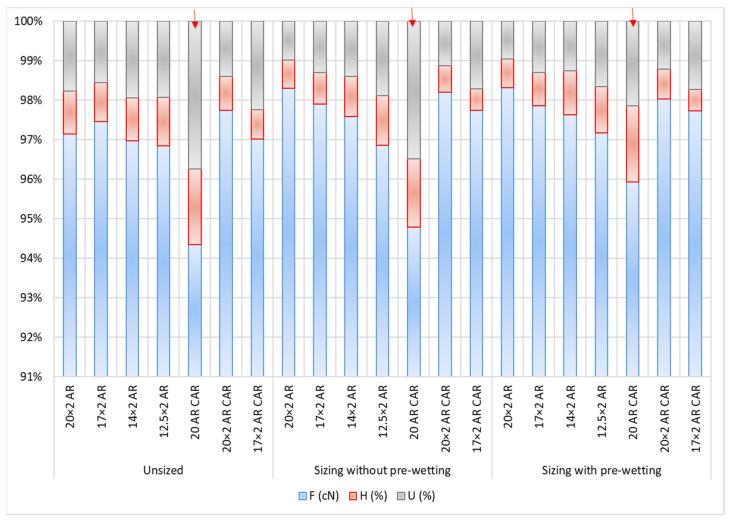
Breaking force (F; cN, hairiness (H; %) and yarn irregularity (U; %) depending on sizing conditions and sample—presence and comparison of individual parameters in unit amount, 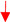
—highlighted differences.

The results of the tests of tensile yarn properties, before and after sizing and with and without pre-wetting, are listed in Table 4, the graphical presentation with error values are shown in Figure 2 and correlation values between breaking forces and elongation at break are shown in Figure 3. Size pick-up, abrasion resistance, yarn irregularity and hairiness before and after sizing, with and without pre-wetting, are presented in Table 5. The comparison of the results of tensile forces and other tested properties (elongation at break, size pick-up, abrasion resistance and hairiness non-uniformity) and their correlated values are shown in Figure 4, Figure 5, Figure 6, Figure 7, Figure 8, Figure 9, Figure 10, Figure 11, Figure 12, Figure 13 and Figure 14.

The breaking properties of the yarn determined on the tensile tester demonstrate an increase in breaking forces of up to 40% after sizing the yarn (Table 4, Figure 4). The coarser plied yarn had higher breaking forces before sizing and this trend continued after sizing. Comparing the yarns according to groups (AR and AR CAR), it can be observed that yarns of the same fineness with the designation AR CAR have a higher breaking force. The carbon content of the yarn contributed to the higher breaking force. Comparing the sized yarn under the sizing conditions, it can be observed that the sized yarn without pre-wetting had higher breaking forces than the sized yarn with pre-wetting, while the elongation at break did not always follow this trend. An exception is the single yarn (20 AR CAR), in which the elongation at break was significantly higher in the sized yarn with pre-wetting. Here, it is possible that the yarn retained more water and void space before sizing due to its greater looseness or openness (larger gaps in the yarn) compared to the plied yarn, so that it absorbed, diluted and retained the size solution in the gaps with a lower percentage of the size solution due to greater diffusion, making the yarn less stiff and allowing for its higher elongation. The finest yarn (12.5 × 2 AR) demonstrated a lower breaking force after sizing with both sizing methods, which is a consequence of the lower size pick-up. This yarn has the smallest number of fibres in the cross-section that under a certain tension and a segment of yarn guiding in the wet state retained less size solution, all of which resulted in a certain irreversible elongation and deformation. An interesting additional effect of this yarn is that the elongation at the break increased significantly after sizing with pre-wetting, which is also a feature of this sizing method. The results of the work of rupture and breaking strength result from the breaking forces and the elongation at break. It can be ascertained that, in addition to the breaking force, the elongation at the break of the sized yarn also increased most frequently after sizing, despite the yarn stiffness (Table 4, Figure 4). The results of the breaking properties obtained demonstrate that the sizing of aramid yarns is justified: the breaking forces increased with sizing (yarn designated with 20 × 2 AR, 20 × 2 AR CAR and 17 × 2 AR CAR stands out), except for the finest yarn designated with 12.5 × 2 AR. The elongation at the break also increased except for yarns designated with 12.5 × 2 AR and 20 AR CAR for sizing without pre-wetting and except for yarn designated with 17 × 2 AR for sizing with pre-wetting.

Figure 5 shows the values of the breaking forces of the tested samples with a pronounced degree of error for 95% certainty. Considering the deviation interval of the breaking forces depending on the sizing conditions and the samples, there is no overlap of the results for the sized yarns, meaning that the breaking forces differ between the samples with 95% certainty.

In the correlation of breaking force and elongation at break (Figure 6, Table 5), a high correlation coefficient can be determined for the unsized yarn (R^2^ = 0.7398), for the sizing without pre-wetting (R^2^ = 0.8623) and for the sizing with pre-wetting (R^2^ = 0.8223), as well as for the total mean values (R^2^ = 0.8045).

The test results: Size pick-up, abrasion resistance, irregularity and hairiness of the yarn per samples and sizing conditions are shown in Table 5 and Figure 7, Figure 8, Figure 9, Figure 10, Figure 11, Figure 12, Figure 13 and Figure 14.

Although all samples were sized at the same time and under the same conditions, the size pick-up changes depending on the yarn type (single yarn and plied), depending on the yarn count (20 to 20 × 2 tex) and depending on the raw material composition (AR, AR + CAR). The finer yarn took up less size pick-up. The yarn designated with AR CAR had a higher size pick-up than the sample designated with AR. The yarn sized without pre-wetting designated with AR and the yarn sized with pre-wetting designated with AR CAR had a higher size pick-up than the yarn without pre-wetting designated with CAR and the yarn with pre-wetting designated with AR (Figure 7, Table 5).

In the correlation of breaking force and size pick-up (Figure 8, Table 5), a high correlation coefficient can be determined for the values obtained by sizing without pre-wetting (R^2^ = 0.8951), by sizing with pre-wetting (R^2^ = 0.8971) and for the overall mean values (R^2^ = 0.8121).

The abrasion resistance (expressed by the number of abrasion cycles to breaking) varies greatly depending on samples and sizing conditions (Figure 9, Table 5). The single yarn designated with 20 AR CAR had the lowest abrasion resistance before and after sizing. For all the yarns, abrasion resistance was not increased by sizing, as in the case of sizing without pre-wetting sample 14 × 2 AR and in the case of pre-wetting all the samples designated with AR and sample 17 × 2 AR CAR. As a rule, the finer the yarn, the lower the abrasion resistance that was recorded after sizing.

Figure 10 shows regression lines, regression equations and correlation coefficients between breaking forces and abrasion resistance. According to the results obtained, a relatively high correlation coefficient can be obtained for the values obtained before sizing (R^2^ = 0.7743), sizing without pre-wetting (R^2^ = 0.8211), sizing with pre-wetting (R^2^ = 0.8707) and the total mean values (R^2^ = 0.7323).

Yarn irregularity differs in the yarn samples before sizing, and this difference decreases in the sample groups AR and AR CAR (Figure 11, Table 5). Higher irregularity was observed in the group of samples designated with AR CAR before and after sizing, especially after sizing without pre-wetting. Yarn irregularity decreased in all samples and sizing conditions in sizing without pre-wetting, except for yarn sample 20 AR CAR. No correlation could be found between the breaking forces and the yarn count with the yarn irregularity.

From the results obtained, it can be noted that the frequency of protruding fibres decreased due to sizing all samples and sizing conditions, except for yarn sample 14 × 2 AR when sizing with pre-wetting (Figure 12, Table 5). The least hairiness was found in yarn designated with 17 × 2 AR CAR before and after sizing, while the highest hairiness was found in the sized single yarn designated with 20 AR CAR.

Figure 13 shows the results of yarn hairiness with a prominent degree of error. The histogram shows the difference among yarn samples as well as among the sizing conditions. Sizing with pre-wetting resulted in a smoother yarn for all yarn samples than sizing without pre-wetting. Taking into account the error coefficient, i.e., the interval of deviation of yarn hairiness under sizing conditions and for the samples, there are some overlaps of results on the unsized and sized yarn in the group of samples AR, which means that yarn hairiness is almost indistinguishable between these samples with a statistical certainty of 95%.

Three parameters: breaking force, hairiness and irregularity in the form of histograms with proportions of the individual values show certain value deviations and mutual differences among the yarn samples (Figure 14). The highlighted bars show the yarn sample designated with 20 AR CAR, which has the highest percentage of hairiness and irregularity and the smallest share of breaking forces, compared to the other yarns. Other yarn types do not show major deviations.

In general, the following can be concluded from the test results (Table 6):

- Size pick-up is higher for yarns designated with AR with sizing without pre-wetting and for yarns designated with AR CAR with sizing with pre-wetting;
- The breaking forces are higher for all samples except for the yarn sample designated with 12.5 × 2 AR with and without sizing;
- The elongation at break is higher for all samples except for the sample 12.5 × 2 AR sized without pre-wetting and the sample 20 AR CAR sized with and without pre-wetting;
- The abrasion resistance of the yarn increased with sizing without pre-wetting, except for sample 14 × 2 AR, while only in two samples abrasion resistance increased with sizing with pre-wetting (20 AR CAR and 20 × 2 AR CAR);
- The yarn irregularity is lower for all samples sized without and with pre-wetting, except for sample 14 × 2 AR, which was sized with pre-wetting;
- The hairiness of the yarn decreased in all samples except for the sample 14 × 2 AR sized with pre-wetting.

## 4. Conclusions

Based on the results obtained, the following can be concluded:

- The finer and single yarn has a lower size pick-up and breaking force;
- The yarn sized without pre-wetting designated with AR has a higher size pick-up and the yarn designated with AR CAR sized with pre-wetting has also a higher size pick-up;
- The results obtained for the breaking properties suggest that the sizing of aramid yarns is justified;
- Sizing increased the breaking forces by up to 40%. A higher breaking force results from the sizing without pre-watering. Increasing breaking forces are recorded in all samples and under sizing conditions, except for the finest sample designated with 12 × 2 AR, which proved to be the most sensitive in wet sizing processing and with a lower size pick-up. The yarns designated with AR CAR with a carbon fibre content had a higher breaking force before and after sizing;
- Elongation at break increased by sizing for the plied yarns except for the finest plied yarn designated with 12.5 × 2 AR sized without pre-wetting and the yarn designated with 17 × 2 AR sized with pre-wetting and except for the single yarn 20 AR CAR sized with pre-wetting;
- The abrasion resistance of the yarn is higher for all samples without pre-wetting, except for sample 14 × 2 AR, which was sized without pre-wetting. The samples sized with pre-wetting mostly demonstrate no increase in abrasion resistance, except for samples 20 AR CAR and 20 × 2 AR CAR, in which abrasion resistance increased. The finer the yarn after sizing, the lower the increase in abrasion resistance;
- The yarn irregularity was reduced by sizing in all samples, especially in yarns sized with pre-wetting, except for sample 20 AR CAR after sizing without pre-wetting. Analysing the samples of the groups AR and AR CAR after sizing, it was found that their mutual differences in irregularity decreased;
- Yarn hairiness or the frequency of protruding fibres due to sizing decreased in all samples and sizing conditions, except for the sample 14 × 2 AR after sizing with pre-wetting.

The second group of yarns with carbon fibre content mostly had better mechanical properties before sizing as well as after sizing. In general, aramid yarn starched with pre-wetting undergoes certain deformations caused by elongation in the wet state and thus reduces the amount of size pick-up, affecting lower breaking forces and strength. Sizing with pre-wetting affected the somewhat greater smoothness of the yarn and its greater regularity. It can be concluded that aramid yarn should be sized with a lower size percentage (up to 4.5%) and without pre-wetting, in order to minimize yarn deformation during sizing and thus improve mechanical properties in the weaving process.

Due to the cost effectiveness of sizing in terms of sizing consumption, sizing with pre-wetting may be preferred, especially for carbon fibre yarns. Single and finer aramid plied yarns require special attention in the sizing process due to their extreme sensitivity to tension in the wet state, in order to avoid negative consequences caused by sizing. This study proved the justification of sizing aramid yarns and yarns blends of aramid and carbon fibres.

## Figures and Tables

**Table 1:** Yarn samples.

Samples	Yarn Designation	Raw Material Composition	Percentage of Raw Materials (%)	Yarn Count (Tex)	Number of Twists (Twist/m)	CV Number of Twists (%)
1	20 × 2 AR	Meta-aramid	95	20 × 2	571.2	2.83
		Para-aramid	5			
2	17 × 2 AR	Meta-aramid	95	17 × 2	624.5	2.56
		Para-aramid	5			
3	14 × 2 AR	Meta-aramid	95	14 × 2	687.1	3.73
		Para-aramid	5			
4	12.5 × 2 AR	Meta-aramid	95	12.5 × 2	723.8	4.25
		Para-aramid	5			
5	20 AR CAR	Meta-aramid	93	20	705.1	3.70
		Para-aramid	5			
		Carbon	2			
6	20 × 2 AR CAR	Meta-aramid	93	20 × 2	564.3	4.11
		Para-aramid	5			
		Carbon	2			
7	17 × 2 AR CAR	Meta-aramid	93	17 × 2	580.2	7.07
		Para-aramid	5			
		Carbon	2			

AR—aramid fibres; CAR—carbon fibres.

**Table 2:** Sizing agents and their percentage in the sizing process.

Size Composition	Industrial Values		Laboratory Values	
	(L/kg)	%	(L/kg)	%
Water (L)	500	88.03	5	88.03
Fibrostin C75 (kg)	50	8.80	0.5	8.80
Inex 773C (kg)	15	2.64	0.15	2.64
Avilor 308AS (kg)	3	0.53	0.03	0.53
Size (L)	568	100	5.68	100

**Table 3:** Sizing conditions during the sizing process.

Sizing Conditions	X	CV (%)
Thread tension between the creel with cross-wound packages and the pre-wetting box	40 cN	16.42
Water temperature in the pre-wetting box	60 °C	7.81
Size solution temperature in the size box	80–85 °C	5.27
Sizing speed	3.2 m/min	11.54
Pressure on the last pair of size solution squeezing rollers	12.5 N/cm^2^	2.51
Temperature of the cylinders of the contact dryer	110 °C	12.00
Moisture of the yarn in the box entry	4%	4.40
Moisture of the yarn in the dryer exit	4%	13.06

X—average value; CV—correlation coefficient (%).

**Table 4:** Breaking forces of the yarn per samples and sizing conditions.

Sizing Conditions	Yarn Designation	Breaking Force/ Differences after Sizing		Elongation at Break/ Differences after Sizing		Work of Rupture		Strength	
		X (cN)	CV (%)	X (%)	CV (%)	X (cN × cm)	CV (%)	X (cN/tex)	CV (%)
Unsized yarn	20 × 2 AR	630.45	6.12	18.28	15.93	44.79	20.52	15.76	6.12
	17 × 2 AR	620.48	4.97	19.04	10.61	45.72	13.76	18.63	4.97
	14 × 2 AR	547.36	7.12	18.49	13.43	38.76	18.53	19.16	7.12
	12.5 × 2 AR	474.98	6.07	19.29	11.83	33.04	16.95	19.00	1.15
	20 AR CAR	338.20	9.04	13.81	26.98	18.73	34.20	16.91	9.04
	20 × 2 AR CAR	774.30	6.71	21.07	9.91	61.18	14.14	19.36	6.71
	17 × 2 AR CAR	629.67	5.50	19.55	10.88	44.46	15.76	18.91	5.50
Sizing without pre-wetting	20 × 2 AR	863.40 +36.95	6.61	22.75 +24.45	11.10	70.10	16.73	21.58	6.61
	17 × 2 AR	688.28 +10.93	6.14	21.43 +12.55	10.97	53.11	16.24	20.67	6.14
	14 × 2 AR	576.84 +5.39	6.21	20.84 +12.71	10.62	42.97	15.64	20.19	6.21
	12.5 × 2 AR	461.15 −2.91	9.10	15.57 −19.28	25.10	28.70	31.56	18.45	9.10
	20 AR CAR	355.96 +5.25	9.15	13.38 −3.11	25.75	19.15	32.31	17.80	9.15
	20 × 2 AR CAR	964.54 +24.57	4.95	22.65 +7.50	19.47	39.15	24.06	34.97	6.95
	17 × 2 AR CAR	800.49 +27.13	6.16	21.49 +9.92	11.94	64.20	15.71	20.01	6.16
Sizing with pre-wetting	20 × 2 AR	805.21 +27.72	6.92	22.02 +20.46	10.20	65.69	15.43	20.26	6.92
	17 × 2 AR	635.92 +2.49	8.34	18.59 −2.36	18.60	45.18	24.46	19.10	8.34
	14 × 2 AR	550.16 +0.52	7.56	20.42 +10.44	13.06	42.32	18.43	19.26	7.56
	12.5 × 2 AR	458.12 −3.55	8.47	15.93 +17.42	22.06	28.98	28.52	18.32	8.47
	20 AR CAR	340.93 +0.81	6.77	16.45 −19.11	16.08	20.92	21.98	17.05	6.77
	20 × 2 AR CAR	866.11 +11.86	4.28	22.35 +6.07	6.84	72.98	9.54	26.01	4.28
	17 × 2 AR CAR	774.82 +23.05	6.89	20.98 +7.31	12.14	58.85	17.35	19.37	6.89

**Table 5:** Size pick-up, abrasion resistance, irregularity and hairiness of the yarn per samples and sizing conditions.

Sizing Conditions	Yarn Designation	Size Pick-Up (Sp/%)		Abrasion Resistance (A/n)		Irregularity (U/%)		Hairiness (H/%)	
		X	CV	X	CV	X	CV	X	CV
Unsized	20 × 2 AR			1487.25	16.78	11.45	15.32	7.11	12.2
	17 × 2 AR			1374.35	16.89	9.87	12.79	6.3	11.45
	14 × 2 AR			1470.05	32.71	10.92	13.65	6.11	11.92
	12.5 × 2 AR			718.55	37.11	9.44	11.86	6	11.86
	20 AR CAR			277.75	31.57	13.42	17.72	6.86	13.79
	20 × 2 AR CAR			1495.95	30.46	11.03	14.81	6.85	11.9
	17 × 2 AR CAR			1501.2	19.12	14.55	20.35	4.78	11.27
Sizing without pre-wetting	20 × 2 AR	4.27	6.55	1508.4	17.27	8.57	10.78	6.31	9.89
	17 × 2 AR	3.5	8.55	1645.65	26.78	9.08	11.54	5.66	10.68
	14 × 2 AR	3.18	9.71	1172.1	18.31	8.22	10.44	6.04	9.11
	12.5 × 2 AR	2.72	5.82	1145.25	38.65	8.98	11.19	5.96	10.87
	20 AR CAR	2.05	8.49	466.95	30.38	13.08	17.73	6.84	11.16
	20 × 2 AR CAR	4.24	10.11	1878.8	15.38	11.11	14.99	6.68	9.99
	17 × 2 AR CAR	3.31	8.21	1744.1	16.89	14	18.84	4.39	8.6
Sizing with pre-wetting	20 × 2 AR	3.45	6.2	1403.2	20.02	7.8	9.87	5.96	8.87
	17 × 2 AR	3.32	5.44	1132.55	17.11	8.45	10.8	5.45	9.7
	14 × 2 AR	3.07	3.91	1025.6	30.4	7.07	9.07	5.3	9.96
	12.5 × 2 AR	2.61	7.09	695.05	21.21	7.8	9.87	5.52	7.7
	20 AR CAR	2.09	7.12	314.4	26.31	7.6	9.48	6.5	9.04
	20 × 2 AR CAR	4.27	4.73	1636.55	26.15	10.72	14.7	5.53	7.93
	17 × 2 AR CAR	4.16	5.29	1037.3	20.21	13.72	18.46	4.23	7.47

Sp—size pick-up (%), A—abrasion resistance (n), n—number of cycles of roller movement along the length of the thread until thread breakage, U—irregularity (%), H—hairiness (%), X—average value and CV—correlation coefficient (%).

**Table 6:** Test results (higher ↑/lower ↓), depending on sizing conditions and yarn samples.

Samp.	Yarn Designation	Size Pick-Up		Breaking Forces		Elongation at Break		Abrasion Resistance		Irregularity		Hairiness	
		A	B	A	B	A	B	A	B	A	B	A	B
1	20 × 2 AR	↑	↓	↑	↑	↑	↑	↑	↓	↓	↓	↓	↓
2	17 × 2 AR	↑	↓	↑	↑	↑	↓	↑	↓	↓	↓	↓	↓
3	14 × 2 AR	↑	↓	↑	↑	↑	↑	↓	↓	↓	↓	↓	↑
4	12.5 × 2 AR	↑	↓	↓	↓	↓	↑	↑	↓	↓	↓	↓	↓
5	20 AR CAR	↓	↑	↑	↑	↓	↓	↑	↑	↑	↓	↓	↓
6	20 × 2 AR CAR	↓	↑	↑	↑	↑	↑	↑	↑	↓	↓	↓	↓
7	17 × 2 AR CAR	↓	↑	↑	↑	↑	↑	↑	↓	↓	↓	↓	↓

A—without pre-wetting, B—with pre-wetting.

## Data Availability

Not applicable.
